# Interleukin-1 and Nuclear Factor Kappa B Signaling Promote Breast Cancer Progression and Treatment Resistance

**DOI:** 10.3390/cells11101673

**Published:** 2022-05-18

**Authors:** Sydney Diep, Mahita Maddukuri, Stephanie Yamauchi, Ganamee Geshow, Nikki A. Delk

**Affiliations:** Biological Sciences Department, The University of Texas at Dallas, 800 West Campbell Road, FO-1, Richardson, TX 75080, USA; sydney.diep@utdallas.edu (S.D.); mahitamaddukuri@gmail.com (M.M.); stephanie.yamauchi@utdallas.edu (S.Y.); ganamee.geshow@utdallas.edu (G.G.)

**Keywords:** interleukin-1, breast cancer, NF-κB

## Abstract

While meant for wound healing and immunity in response to injury and infection, inflammatory signaling is usurped by cancerous tumors to promote disease progression, including treatment resistance. The interleukin-1 (IL-1) inflammatory cytokine family functions in wound healing and innate and adaptive immunity. Two major, closely related IL-1 family members, IL-1α and IL-1β, promote tumorigenic phenotypes and contribute to treatment resistance in cancer. IL-1 signaling converges on transactivation of the Nuclear Factor Kappa B (NF-κB) and Activator protein 1 (AP-1) transcription factors. NF-κB and AP-1 signaling are also activated by the inflammatory cytokine Tumor Necrosis Factor Alpha (TNFα) and microbe-sensing Toll-Like Receptors (TLRs). As reviewed elsewhere, IL-1, TNFα, and TLR can promote cancer progression through NF-κB or AP-1. In this review, we focus on what is known about the role of IL-1α and IL-1β in breast cancer (BCa) progression and therapeutic resistance, and state evidence for the role of NF-κB in mediating IL-1-induced BCa progression and therapeutic resistance. We will present evidence that IL-1 promotes BCa cell proliferation, BCa stem cell expansion, angiogenesis, and metastasis. IL-1 also regulates intracellular signaling and BCa cell hormone receptor expression in a manner that confers a growth advantage to the tumor cells and allows BCa cells to evade therapy. As such, the IL-1 receptor antagonist, anakinra, is in clinical trials to treat BCa and multiple other cancer types. This article presents a review of the literature from the 1990s to the present, outlining the evidence supporting a role for IL-1 and IL-1-NF-κB signaling in BCa progression.

## 1. Introduction

**Figure 1:**
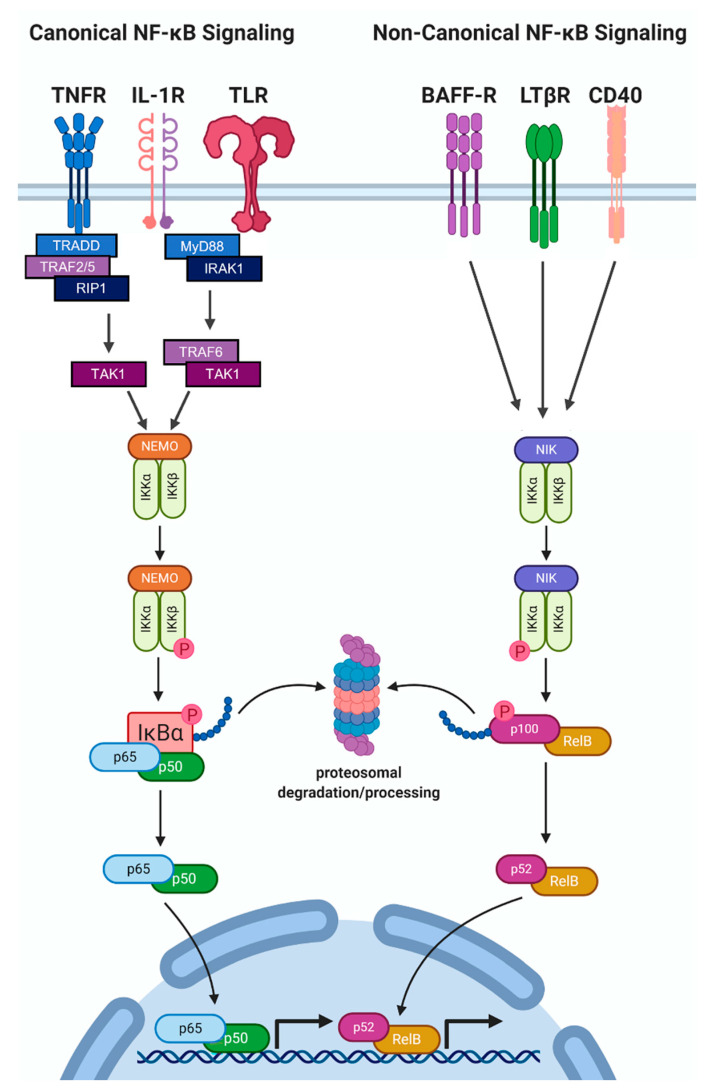
NF-κB signaling. Canonical IL-1 signaling is induced by molecules such as IL-1, TNF, or LPS binding to their respective receptors IL-1R (Interleukin-1 Receptor), TNFR (Tumor Necrosis Factor Receptor), or TLR (Toll-like Receptor). Ligand–receptor interaction recruits Myeloid Differentiation Primary Response 88 (MyD88) adaptor protein and IL-1 Receptor-Associated Kinase 1 (IRAK1) to the IL-1R or TLR receptors, leading to TNF Receptor-Associated Factor 6 (TRAF6)/TGFβ-Activated Kinase (TAK1)/IκB Kinase (IKK; IKKα/IKKβ/NEMO complex) activation and subsequent degradative phosphorylation of the Nuclear Factor Kappa B (NF-κB) inhibitor, IκBα. Cytoplasmic NF-κB (p65/p50) is then freed from IκBα binding to translocate to the nucleus to activate transcription. TNF receptor-associated death domain (TRADD), receptor-interacting protein kinase 1 (RIP1), and TRAF2 or TRAF5 (TRAF2/5) are recruited to TNFR to activate TAK1/IKK, leading to IκBα degradation and NF-κB nuclear translocation. In non-canonical NF-κB signaling, molecules such as BAFF, CD40 ligand, or Lymphotoxin Beta bind to their respective receptors, B-cell Activating Factor Receptor (BAFF-R), Cluster of Differentiation 40 (CD40), or Lymphotoxin Beta Receptor (LTβR) to activate NF-κB-inducing Kinase (NIK), which phosphorylates and activates IKKα. Activated IKKα phosphorylates p100, leading to p100 ubiquitination and proteolytic cleavage into the p52 subunit. The RelB/p52 heterodimer is then able to translocate to the nucleus and activate gene transcription. Image created using BioRender.

The interleukin-1 (IL-1) family of cytokines plays a key role in initiating and regulating inflammatory and immune responses. The IL-1 family consists of eleven members that include pro- and anti-inflammatory cytokines and receptor antagonists [1]. Two defining IL-1 family members encoded by distinct genes, IL-1 alpha (IL-1α) and IL-1 beta (IL-1β), each bind to the type 1 IL-1 receptor (IL-1R1) and have similar biological activity [1]. IL-1α or IL-1β binding to IL-1R1 induces IL-1R1 dimerization with the IL-1R accessory protein (IL-1RAcP) [2,3]. The resulting complex then recruits the signaling adaptor protein Myeloid Differentiation Primary Response 88 (MyD88) which, in turn, recruits IL-1 Receptor-Associated Kinase 1 (IRAK1) [2,3]. IRAK1 activates the TNF Receptor-Associated Factor 6 (TRAF6)/TGFβ-Activated Kinase (TAK1)/IκB Kinase (IKK; IKKα/IKKβ/NEMO complex) signaling cascade that leads to phosphorylation and degradation of the Nuclear Factor Kappa B (NF-κB) inhibitor, IκBα, thereby freeing cytoplasmic NF-κB to translocate to the nucleus to activate transcription of inflammatory and immune genes [2,3] (Figure 1).

IL-1 is produced and secreted by epithelial, endothelial, platelet, fibroblast and immune cells (e.g., macrophages and neutrophils) to protect against injury and infection as part of innate and adaptive immune responses [4]. However, uncontrolled or unresolved inflammatory signaling leads to chronic inflammation, which can drive cancer initiation and progression [5]. In the tumor microenvironment, cancer cells are also a source of inflammatory cytokines and, in turn, exacerbate tumor-promoting inflammation [5]. As reviewed below, IL-1 and NF-κB accumulation and activity in the tumor microenvironment are clinically significant and promote breast cancer (BCa) progression and therapeutic resistance.

## 2. IL-1 Expression and Signaling Is Associated with Breast Cancer (BCa) Disease Progression

### 2.1. IL-1 Accumulation Is Elevated in BCa

It is well established that IL-1α [6,7,8,9,10] or IL-1β [6,11,12,13,14,15] levels are elevated in primary tumors or serum of BCa patients and correlate with aggressive disease phenotypes such as advanced stage, basal subtype, or metastasis. Immunohistochemistry (IHC) and enzyme-linked immunosorbent assay (ELISA) revealed that IL-1α and IL-1β are elevated in diseased versus normal adjacent or healthy breast tissue or serum [8,11,13,14,15] and immunological multiplex analysis of primary BCa tissue from 149 patients showed that IL-1β levels increase with advancing stage [11]. Breast cancer subtypes are classified as luminal A (Estrogen Receptor Alpha (ERα) positive, Progesterone Receptor (PR) positive, Erb-B2 receptor tyrosine kinase 2 (HER2) negative), luminal B (ERα+/PR+/HER2+), HER2+, or basal (ERα-/PR-/HER2-; triple negative BCa) [16]. Basal or triple negative BCa (TNBC) lacks the therapeutic targets, ERα and PR hormone receptors and HER2, leading to poor outcome [16]. ELISA of serum from 200 BCa patients revealed that IL-1α and IL-1β are higher in hormone receptor negative patients versus hormone receptor positive patients [6]. In support of these findings, other studies showed that IL-1α or IL-1β levels are inversely correlated with ERα and/or PR levels in BCa tumor tissue from primary or regional metastasis [9,10,13]. IHC of 136 primary BCa tumor tissues performed by Liu and colleagues revealed that elevated IL-1α protein levels positively correlate with HER2+ status [7]. Elevated IL-1α was also found to be associated with cancer stem cell (CSC) accumulation and poor metastasis-free survival in BCa patients [7]. In kind, IHC of 1189 primary BCa tumor tissues showed that elevated IL-1β is positively associated with patient relapse and metastasis [12]. Taken together, high IL-1 accumulation is clinically relevant in BCa and is associated with disease progression.

### 2.2. NF-κB Protein Accumulation and Activity Are High in Hormone Receptor Negative BCa

The NF-κB transcription factor is a heterodimer comprised of DNA-binding, transactivating subunits, RELA (p65), RELB, or c-REL, dimerized with DNA-binding subunits, NF-κB1 (p105/p50) or NF-κB2 (p100/p52) [17]. In canonical NF-κB signaling, RELA/p50 or c-REL/p50 heterodimers translocate to the nucleus in response to IL-1, TNFα, or bacterial lipopolysaccharide (LPS) activation of TAK1/IKK-induced phosphorylation and degradation of IκBα [17,18,19,20,21]. Non-canonical NF-κB signaling is mediated by the NF-κB-inducing Kinase (NIK)/IKKα complex phosphorylation and nuclear translocation of RELB/p52 in response to B-cell Activating Factor (BAFF), Cluster of Differentiation 40 (CD40) ligand, or Lymphotoxin Beta [17,22,23,24,25] (Figure 1).

As observed for IL-1, elevated NF-κB accumulation is associated with aggressive clinicopathological BCa phenotypes. As stated above, ERα- and TNBC tumors lack the ERα therapeutic target, making these tumors endocrine-resistant and limiting treatment options for these BCa patients [16,26,27]. Like IL-1, NF-κB accumulation and activity were found to be elevated in hormone receptor negative BCa tumors. For example, Biswas et al. performed Electrophoretic Mobility Shift Assay (EMSA) of nuclear extracts from BCa patient tissue and found that NF-κB (RELA, c-REL, p50) DNA-binding activity is comparatively high in ERα^−^ tumors [28]. In addition, Zhou and colleagues found that NF-κB (RELA and/or p50) DNA binding activity was higher in the ER low BCa tissue and correlated with tamoxifen resistance, metastatic relapse, and poor disease-free survival in BCa patients [29]. Finally, elevated non-canonical NF-κB (*RELB* and *NFKB2*) gene expression and nuclear accumulation are inversely correlated with ERα+ nuclear accumulation and are associated with relapse and poor disease-free survival for BCa patients [30]. This indicates that IL-1-independent NF-κB activity is also clinically significant.

Functionally, NF-κB has been shown to repress ERα accumulation leading to endocrine resistance. An estrogen-independent MCF7 BCa cell subline acquired NF-κB-dependent proliferation, invasion, tumor growth, and tamoxifen resistance concomitant with the loss of ERα accumulation [31]. Treatment of the ERα- MCF7 subclone with the IKKβ inhibitor, IMD-0354, restored ERα levels and tamoxifen sensitivity, suggesting that canonical NF-κB signaling represses ERα accumulation to promote endocrine resistance [31]. RELB was found to signal Blimp1 (B-lymphocyte-induced maturation protein)-dependent repression of *ERα* expression in MCF7 and ZR-75-1 BCa cell lines [32]. Thus, canonical and non-canonical NF-κB signaling repress ERα accumulation. 

IL-1 also represses hormone receptors [33,34]. Although IL-1 levels were not assessed alongside NF-κB accumulation or activity in the studies above, we have found that RELA mediates IL-1 repression of *Androgen Receptor* (*AR*) hormone receptor expression in prostate cancer cells (PCa) [35] and, thus, NF-κB activity likely mediates IL-1 repression of *ERα* expression in BCa cells.

## 3. The Role of IL-1-NF-κB in BCa Progression

As discussed below, IL-1 and NF-κB signaling promote BCa tumor growth and progression by supporting tumor cell proliferation, clonogenicity and stemness, angiogenesis, epithelial to mesenchymal transition (EMT), dissemination of circulating tumor cells (CTCs), invasion, and metastasis. IL-1 is produced by multiple different cell types in the primary and metastatic niches, including cancer cells, macrophages, and osteoblasts to promote tumorigenic phenotypes such as chemotaxis, angiogenesis, and metastatic niche colonization. IL-1 paracrine and autocrine signaling are dynamic, context-specific, and, consequently, pro-tumorigenic in BCa.

### 3.1. Proliferation

Acute inflammation is anti-tumorigenic [36,37], and we and others have shown that acute exposure (e.g., within 9 days) to IL-1 is cytotoxic and cytostatic for epithelial cancer cell lines, including BCa cell lines [35,38,39,40,41]. Using a PyMT/*IL-1R1*^−/−^ and PyMT/*IL-1α*^−/−^ mouse model, Dagenais et al. found that IL-1R1- or IL-1α-deficient mice develop mammary tumors at a significantly faster rate with increased cell proliferation in the early stages of tumor development [42]. The authors conclude that IL-1 represses early mammary tumorigenesis [42]. Noting that they are modeling acute IL-1 inflammation, Tulotta and colleagues found that anti-tumor immune cells were recruited to the syngeneic tumors of IL-1β^−/−^ mice orthotopically injected with *IL-1β*-overexpressing luminal B E0771 BCa cells [43]. The authors speculate that the anti-tumor immune cell infiltration led to the reduced tumor growth observed within seventeen days post BCa cell injection [43]. Thus, acute IL-1 exposure can reduce BCa cell proliferation and viability and remodel the immune component of the tumor microenvironment to favor tumor repression. Together, these studies support an anti-tumorigenic role for acute IL-1 inflammation [36].

While IL-1 can be cytotoxic and cytostatic, IL-1 can also promote cancer cell proliferation. Cancer is an aging-related disease where chronic inflammation caused by resident senescent fibroblasts can cause tumor initiation and progression [44]. The anti-inflammatory flavonoid, apigenin, inhibits IL-1α-NF-κB signaling in senescent fibroblasts and was shown to reduce inflammatory cytokine secretion and paracrine induction of MDA-MB-231 TNBC and luminal ZR-75-1 BCa cell proliferation [45]. Thus, IL-1α-NF-κB signaling in senescent fibroblasts produces paracrine factors that promote BCa proliferation. To test the hypothesis that bone remodeling cytokines, such as IL-1β, can stimulate BCa proliferation in the bone, Sosnoski et al. co-cultured osteoblasts in 3D with a non-metastatic MDA-MB-231 TNBC subline or luminal A MCF7 cells in the presence of exogenous IL-1β [46]. In the absence of IL-1β the BCa cells remained dormant, but the addition of IL-1β induced BCa cell proliferation [46]. These results suggest that there is a bone metastatic niche-specific milieu of paracrine factors or cell types needed to support IL-1 pro-tumorigenic functions. Indeed, Tulotta et al. reported that while IL-1β can be anti-tumorigenic in the primary tumor [43], IL-1β can promote osteoclast activity in bone leading to larger osteolytic BCa lesions [12]. Together, these studies indicate that IL-1 induces context-specific BCa tumor repression or growth that is influenced by the make up of the tumor microenvironment.

### 3.2. Clonogenicity/Stemness

In addition to inducing proliferation, IL-1 is implicated in promoting cancer cell stemness. Cancer stem cells (CSC) are self-renewing, tumorigenic, and resistant to chemoradiation [47]. Liu et al. demonstrated that treatment of normal mammary epithelial MCF10A cells with IL-1α or IL-1β increases clonogenicity [7]. They further discovered that *HER2* overexpression drives autocrine IL-1α-NF-κB-dependent CSC expansion in MCF10A cultures in vitro and loss of IL-1α signaling in *MMTV-Her2/IL-1α^+/−^* and *MMTV-Her2/IL-1α^−/−^* mice reduces CSC accumulation in primary tumors [7]. Eyre and colleagues used ERα^+^ and ERα^−^ BCa patient-derived tumors and cell lines to determine the effect of IL-1 on cancer stem cell-like phenotypes. They found that bone marrow-derived IL-1β activates BCa cell autocrine Wnt signaling via NF-κB and cAMP Response Element-Binding Protein (CREB) activation, which led to BCa cell clonogenicity in vitro and BCa cell metastasis and bone colonization in vivo [48]. Aldehyde dehydrogenase (ALDH) is a marker for BCa stem cells; ALDH^+^/IL-1R1^+^ CSCs are enriched in anti-estrogen-treated BCa patients [49]. Furthermore, ALDH^+^/IL-1R1^+^ BCa stem cells are tumor-initiating and show enhanced clonogenicity that can be attenuated with anakinra [49]. Taken together, IL-1-NF-κB contributes to BCa tumorigenicity by promoting CSC accumulation and clonogenicity. 

### 3.3. Angiogenesis

IL-1 promotes tumor angiogenesis by upregulating Vascular Endothelial Growth Factor (VEGF) signaling in tumor cells and endothelial cells and by regulating endothelial cell function. Murine 4T1 BCa cells treated with leptin in the presence of mouse anti-IL-1R antibody showed reduced VEGF and VEGF Receptor (VEGFR) mRNA and protein levels [50]. C-X-C chemokine receptor type 7 (CXCR-7) induces endothelial progenitor cell (EPC) *VEGF* expression and tube formation; treatment with the IKKα/IKKβ inhibitor, BAY 11-7082, blocked IL-1β-NF-κB-dependent CXCR-7 upregulation in EPCs [51]. IL-1Ra or anakinra reduced tumor angiogenesis in vivo, where IL-1Ra inhibited proliferation of the HUVEC endothelial cell line in vitro and inhibited MCF7 BCa xenograft tumor growth and vascularization in vivo [52,53]. Finally, *IL-1α* and *IL-1β* knockout mice showed reduced vascularization and growth of murine DA3 BCa tumors [54]. Taken together, IL-1-NF-κB is sufficient to induce angiogenesis.

### 3.4. Metastasis

IL-1 has a well-established role in promoting BCa metastasis. For example, *HER2*-overexpressing BCa cells show IL-1α-dependent tumor growth and lung metastasis in vivo [7]. Bone-engrafted humanized mouse models injected orthotopically with MDA-MB-231 TNBC or *IL-1β*-overexpressing MDA-MB-231 cells and treated with anakinra or IL-1 neutralizing antibody, canakinumab, revealed that IL-1 enriches circulating tumor cells (CTCs) and bone metastasis [12,55]. CTCs are disseminated tumor cells that can establish metastases [56] and the Ottewell lab discovered that CTCs and bone metastases have high levels of IL-1β and express EMT markers [12]. In kind, inhibition of *IL-1β* expression and signaling by the triterpene, celastrol [57], or the sesquiterpene, zerumbone [58], correlates with reduced cell viability and/or motility in TNBC cell lines. Transwell and conditioned medium experiments using MDA-MB-231 BCa cells and human bone fragment from elective hip replacement surgery revealed that bone marrow-derived IL-1β may serve as a chemoattractant for BCa cell bone metastasis and colonization [59]. Thus, IL-1 promotes metastasis by inducing EMT, motility, CTC accumulation, and metastatic site colonization.

IL-1 has been shown to promote BCa invasion, migration, and metastasis through NF-kB signaling. For example, the inhibition of NF-kB activation with the IKKα/IKKβ, Bay11-7082, attenuated IL-1β-induced migration of MDA-MB-231 TNBC cells under hypoxic conditions [60]. Treatment of *Transglutaminase 2* (*TG2*)-overexpressing MCF7 cells with IL-1β and Bay11-7082 demonstrated that IL-1β-NF-κB mediates TG2 induction of interleukin-6 (IL-6) [61]. Notably, the TG2/IL-1β/IL-6 axis causes BCa growth and metastasis in vivo and elevated TG2/IL-6 co-expression is correlated with poor metastasis-free survival in BCa patients [61,62]. N-acetyltransferase 1 (NAT1), which is overexpressed in luminal BCa cell lines and patient tissue and correlates with BCa bone metastasis, was shown to induce NF-κB-dependent IL-1β expression and secretion in BCa cells and to promote BCa bone metastasis and establishment of the bone metastatic niche [63]. Conversely, the repression of NF-κB activity by the sesquiterpene, zerumbone, inhibited *IL-1β* expression in TNBC cells, thereby blocking TNBC invasion in vitro [58]. Finally, apigenin blocked senescent fibroblast-induced BCa cell invasion and EMT marker accumulation, inferring that IL-1α-NF-κB promotes BCa metastasis [45]. Notably, NF-κB signaling gene signatures were found to be associated with the presence of mesenchymal-like CTCs in primary BCa tumors [64], suggesting NF-κB signaling is an early event in the onset of metastasis. Taken together, IL-1-NFκB signaling functions in a feedforward loop to promote metastasis. 

Once at the metastatic niche, IL-1-NF-κB signaling supports BCa cell colonization and tumor growth. BCa cells that invade the lung establish the metastatic niche by secreting IL-1α and IL-1β to induce NF-κB-dependent C-X-C Motif Chemokine Ligand 9/10 (CXCL9/10) production in lung fibroblast [65]. Fibroblast-secreted CXCL 9/10 binds the C-X-C Motif Chemokine Receptor 3 (CXCR3) on the invading BCa cells to promote BCa lung colonization and tumor growth [65]. The anti-inflammatory flavonoid, apigenin, inhibits IL-1α-NF-κB signaling and cell invasion and reduces CXCL10 secretion from lung fibroblast [45], supporting a role for IL-1α in establishing the BCa metastatic niche in lung. 

In addition to NF-κB, other signaling molecules are known to mediate IL-1-induced BCa metastasis. Using *Focal Adhesion Kinase* (*FAK*) knockout mouse fibroblast and *FAK* siRNA in MCF7 cells, Mon et al. demonstrated that FAK/proto-oncogene tyrosine-protein kinase (Src) signaling mediates IL-1-induced MMP9 secretion and cell invasion [66]. Franco-Barraza et al. found that IL-1β activates phosphatidylinositol 3-kinase (PI3K)/Rac Family Small GTPase 1 (Rac1)-dependent cell motility in MCF7 cells, which can be blocked with IL-1Ra [67]. Using a highly IL-1-responsive MCF7 subclone, the same group discovered that novel IL-1β signaling through β-catenin transactivation induces BCa cell line proliferation, up-regulation of EMT genes, motility, and invasion [68]. NF-κB was shown to signal Tropomodulin 1 (TMOD1)-dependent β-catenin transactivation and MMP secretion in TNBC BCa cells leading to invasion and tumor growth [69]. IL-1β also promotes matrix metalloproteinase (MMP) secretion to facilitate BCa invasion [57,67]. Ito-Kureha and colleagues showed that TNFα stimulates NF-κB transcription of *TMOD1* [69], suggesting that IL-1 may also transactive β-catenin and induce MMP secretion via the NF-κB-TMOD1 axis. These data show that there are multiple different parallel and integrated pathways governing IL-1-induced metastasis.

## 4. The Role of IL-1 in BCa Therapeutic Resistance

### 4.1. Endocrine Resistance

The foremost studied culprit in BCa tumor initiation and progression is ERα. ERα is a nuclear hormone receptor that promotes BCa cell proliferation and survival and is overexpressed in over 70% of diagnosed BCa [70]. Thus, ERα-targeting therapies that block estrogen production or directly inhibit ERα activity are standard of care [71]. While 50–70% of ERα^+^ BCa patients successfully respond to ER-targeting therapies, 30–50% of ERα^+^ BCa patients have innate or acquired resistance to ER-targeting therapies (i.e., endocrine resistance) [27]; 15–30% of patients that acquire treatment resistance develop tumors with low or no *ERα* gene expression [27,70,72]. Not surprisingly, ERα low/negative (ERα^low/−^) tumors are predictive of BCa patient endocrine resistance and poor prognosis as compared to BCa patients with higher ERα tumor levels [73,74,75,76,77]. Finally, ERα-targeting therapies are also ineffective on TNBC, which comprises 15–20% of diagnosed BCa patients [78].

IL-1 can support endocrine resistance by causing the de novo accumulation of ERα^low/−^ cells or by conferring resistance against anti-estrogens. We [33] and others [38,79] found that acute IL-1α or IL-1β exposure represses ERα and PR mRNA and protein accumulation in breast cancer cell lines. Jiménez-Garduño et al. found that IL-1β repression of *ESR1* expression can occur through Twist Family BHLH Transcription Factor 1 (TWIST1)-dependent methylation of the *ESR1* locus [79]. We find that concomitant with hormone receptor repression, IL-1 induces upregulation of pro-survival genes that are basally high in TNBC cell lines [33,80]. Therefore, acute IL-1 exposure may confer a growth advantage and enable BCa cells to evade anti-androgen therapy. Indeed, Jiménez-Garduño and colleagues showed that a highly IL-1β-responsive MCF7 subline had reduced sensitivity to tamoxifen anti-estrogen cytotoxicity when treated with IL-1β; and tamoxifen insensitivity was reversed by inhibiting the TWIST1 signaling cascade [79]. MCF7 cells treated chronically with IL-1β plus estradiol also developed tamoxifen resistance concomitant with elevated gene expression of the *MRP2* multidrug resistant ABC transporter and *RELA* [81], suggesting IL-1-NF-κB promotes endocrine and drug resistance. In kind, the IKKβ inhibitor, parthenolide, sensitized HER2+ BT474 and *HER2*-overexpressing MCF-7 BCa cell lines to tamoxifen [82], suggesting that the HER2-IL-1-NF-κB axis promotes endocrine resistance.

Gene expression analysis of ERα+ metastatic BCa patients showed that IL-1 signaling is activated in ALDH+ cancer stem cells (CSC). *IL-1R1* expression was increased in ALDH+ BCa cells following tamoxifen or fulvestrant treatment and anakinra attenuated the clonogenicity of tamoxifen- or fulvestrant-resistant MCF7 sublines [49]. Thus, IL-1 confers anti-estrogen resistance. One possible mechanism is that dormant ALDH^+^/IL-1R1^+^ CSCs can evade anti-estrogen therapy and re-enter the cell cycle at a later time to form recurrent tumors [49].

When comparing tamoxifen and fulvestrant, Abrahamsson and colleagues found that fulvestrant is more effective than tamoxifen at blocking IL-1-associated phenotypes. Fulvestrant-treated mice orthotopically injected with MCF7 cells or PyMT murine mammary cancer cells formed tumors that showed less macrophage and neutrophil infiltration and less angiogenesis than tamoxifen-treated mice; and in a zebrafish metastasis model, the tumors showed less BCa cell dissemination [83]. Fulvestrant was also found to reduce cytokine secretion, including IL-1β secretion, from monocytes [83]. Taken together, IL-1 supplied by tumor-infiltrating immune cells may be sufficient to provide a greater growth advantage for BCa tumors in the presence of tamoxifen versus fulvestrant anti-estrogen.

The estrogen-independent ERα- HMC1-8 BCa cell line and an estrogen-independent ERα− MCF7 subline showed reduced proliferation, clonogenicity, ERα protein re-expression, and/or restored sensitivity to tamoxifen when *RELA* was silenced or cells were treated with the IKKβ inhibitor, IMD-0354 [31]. Tamoxifen sensitivity was also restored in two estrogen-independent, ERα+ MCF7 sublines, MCF7/RR and MCF7/LCC9, when RELA/p65 was pharmacologically inhibited [84]. The IKKβ inhibitor, parthenolide, restored fulvestrant sensitivity in MCF7/LCC9 cells [85]. Finally, MCF7 cells overexpressing AKT required constitutive NF-κB activity to acquire tamoxifen resistance [86]. These studies show that the constitutive NF-κB activity acquired in response to stimuli such as estrogen deprivation leads to anti-estrogen resistance [87]; it follows that chronic IL-1-NF-κB signaling in BCa cells would, likewise, confer similar anti-estrogen resistance.

### 4.2. Chemotherapy Resistance

Chemotherapy upregulates IL-1 expression in immune and BCa cells [88,89]. In kind, IHC of tumors from 51 BCa patients pre- and post-anthracycline- and/or taxane-based neoadjuvant chemotherapy showed that nuclear RELA/p65 accumulation is predictive of therapeutic resistance, suggesting that NF-κB activity promotes chemoresistance [90]. Buchholz et al. came to the same conclusion performing IHC analysis for nuclear NF-κB staining on 82 BCa tumor tissues from patients treated with neoadjuvant 5-fluorouracil, doxorubicin, and cyclophosphamide [91]. Together, this suggests a vicious cycle between chemotherapy and inflammation that sets the stage for IL-1-NF-κB-induced chemoresistance.

Having established that *HER2* overexpression drives IL-1α-NF-κB-dependent CSC expansion and IL-1α-dependent tumor growth and lung metastasis, Liu et al. further demonstrated that the genetic loss of IL-1α in the HER2^+^/ERα^−^/PR^−^ HCC1954 BCa cell line sensitizes the BCa cells to cisplatin or paclitaxel cytotoxicity [7]. Pharmacological inhibition of IL-1α-NF-kB signaling in orthotopic HCC1954 xenografts synergistically reduced tumor size in mice treated with paclitaxel [7]. Interestingly, paclitaxel increased clonogenicity in vitro and CSC expansion in vivo, both of which were attenuated by IL-1α inhibition [7]. Using a highly IL-1β-responsive MCF7 subclone, Meza’s group demonstrated that IL-1β attenuates doxorubicin cytotoxicity [92]. IL-1β-induced resistance was dependent on Baculoviral Inhibitor of Apoptosis Protein Repeat Containing 3 (BIRC3), which is a member of the Inhibitor of Apoptosis (IAP) protein family [92]. Thus, IL-1-NF-κB promotes BCa chemoresistance.

### 4.3. Immunotherapy and Immunosuppression

While immunotherapy may be a promising treatment option for TNBC patients [93], IL-1 has been shown to be immunosuppressive and, thus, could limit immunotherapy efficacy for BCa patients. Syngeneic mouse models have demonstrated that IL-1 supports an immunosuppressive tumor microenvironment (TME). For example, IL-1Ra treatment of mice harboring orthotopic EO771 mammary tumor cells reduced the infiltration of immunosuppressive myeloid-derived suppressor cells (MDSCs) and tumor-associated macrophages (TAMs) [94]. Likewise, orthotopic injection of 4T1 BCa cells into *IL-1β* knockout mice formed tumors that regressed, had reduced immunosuppressive macrophage infiltration and activation, and favored anti-tumor dendritic cell infiltration [95]. Finally, using 16 distinct genetically engineered BCa mouse models, de Visser’s group discovered that p53-null BCa cells secrete Wnt ligand which activates macrophages to produce IL-1β, leading to an increase in systemic neutrophil circulation and BCa metastasis [96]. In their model, systemic neutrophil circulation is immunosuppressive, thereby supporting BCa metastasis [96]. Taken together, IL-1 promotes an immunosuppressive TME. As such, treatment of wild type mice with anti-IL-1β and anti-PD-1 was able to synergistically inhibit tumor progression [95], suggesting that adjuvant IL-1 inhibition could improve BCa patient response to immunotherapy. 

## 5. Anakinra Clinical Trials

As reviewed by Wang et al. [97], there are many broad-spectrum drugs, such as proteasome inhibitors (e.g., Bortezomib, Carfilzomib), histone deacetylase inhibitors (e.g., Vorinostat, Romidepsin), and natural compounds (e.g., curcumin, resveratrol) that block constitutive NF-κB activity and are the subject of various cancer clinical trials, including current BCa clinical trials (NCT04265872, NCT01142401, NCT02257476, NCT00616967, NCT03742245, NCT02393794, NCT03980509). However, NF-κB-specific drug inhibitors that target NF-κB subunits (SN-50/52, NBD), prevent NF-κB DNA binding (PBS-1068, parthenolide), or block IκBα degradation (IMD-0354, BAY-11-7082, PS-1145) have not yet translated to clinical trials for cancer [97]. Given the IL-1 target specificity of anakinra and its FDA approval, anakinra is a promising therapy to specifically block IL-1-NF-κB-induced BCa tumorigenicity.

Anakinra, a recombinant human IL-1Ra, is FDA-approved for rheumatoid arthritis and is the subject of several cancer clinical trials, including BCa (Table 1). Multiple animal studies have shown the effectiveness of anakinra against BCa tumor growth and progression, including the inhibition of cell proliferation, angiogenesis and metastasis [11,12,43,48,52,53,55]. Importantly, anakinra is a novel therapeutic against BCa tumors resistant to conventional therapies. For example, anakinra has been shown in preclinical models to be effective against TNBC tumor growth. Anakinra reduced tumor volume in humanized NOD/SCID mice injected subcutaneously with the Hs578T TNBC cell line [11]. Treatment with anakinra pre- and post-intravenous injection of a bone-homing TNBC cell line derivate, MDA-MB-231-IV, reduced tumor cell proliferation, tumor angiogenesis, and bone colonization in nude mice [53]. Finally, IL-1β-neutralizing antibody or anakinra was sufficient to block in vivo metastasis and bone colonization of a spontaneous bone metastatic luminal A patient-derived xenograft and the bone-homing derivative, MDA-MB-231_BH [48]. Thus, anakinra is effective against basal and luminal BCa subtypes.

O’Shaughnessy and colleagues conducted a phase I clinical trial (NCT01802970) to assess the safety of anakinra plus chemotherapy for metastatic BCa patients. The reported results find that anakinra in combination with chemotherapy is safe and the analysis of whole blood transcripts showed a reduction in IL-1 and Toll-like Receptor (TLR)-associated signaling and upregulation of PD-L1 and immunosurveillance signaling [98]. Thus, IL-1 inhibition may make BCa tumors more susceptible to immunotherapy. In multiple myeloma [99] and colorectal cancer [100] phase II clinical trials, anakinra in combination with chemotherapy increased patient survival. Taken together, the IL-1 inhibitor, anakinra, is a promising novel therapeutic to treat BCa disease across subtypes, including TNBC which has no targeted therapy.

## 6. Conclusions

**Figure 2:**
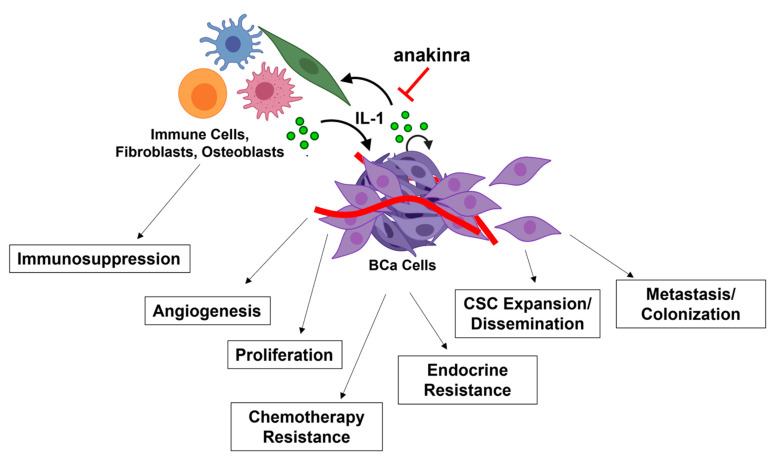
IL-1 promotes BCa tumor progression and treatment resistance. IL-1 is secreted by cells of the primary and metastatic tumor microenvironment, such as tumor cells, immune cells, fibroblasts, and osteoblasts to activate canonical, intracellular NF-κB signaling. IL-1 autocrine and paracrine signaling induce breast cancer (BCa) cell proliferation, tumor angiogenesis, cancer stem cell (CSC) expansion and dissemination, tumor cell metastasis, and tumor cell colonization of the metastatic niche. In addition to promoting tumorigenic phenotypes, IL-1 supports BCa therapeutic resistance, including endocrine resistance, chemotherapeutic resistance, and resistance to immunotherapy. The IL-1 inhibitor, anakinra, can reverse IL-1-induced BCa tumorigenicity and therapeutic resistance. Image created using BioRender.

IL-1 is a pleiotropic inflammatory cytokine that functions in immune response and wound healing. However, cells of the tumor microenvironment usurp IL-1 functions to promote disease progression, including therapeutic resistance. IL-1 is secreted by cells in the tumor stroma and cancer cells. IL-1 paracrine and autocrine signaling lead to cancer cell proliferation, endothelial cell angiogenesis, immune cell-mediated immunosuppression, cancer stem cell enrichment and dissemination, and fibroblast- and osteoblast-supported metastatic niche colonization (Figure 2). IL-1 tumorigenic phenotypes provide a growth advantage to cancer cells that leads to disease progression and therapeutic resistance. In BCa, the IL-1 tumorigenic phenotypes underlie endocrine resistance, chemotherapy resistance and immunosuppression. As such, anakinra is a promising therapeutic inhibitor of IL-1 signaling that is currently under clinical trial investigation for multiple cancer types, include BCa. 

As a well-established mediator of IL-1 signaling, when investigated, studies confirm that NF-κB mediates IL-1-induced tumorigenic phenotypes in BCa. However, IL-1-independent, non-canonical, and/or constitutive NF-κB activity also promote and correlate with BCa progression. For example, constitutive NF-κB signaling causes endocrine resistance in BCa cells. However, while the model systems demonstrating NF-κB-dependent treatment resistance are derived from estrogen-independent BCa cell lines, one can extrapolate that canonical NF-κB signaling also mediates IL-1-induced treatment resistance. Therefore, it is imperative to develop efficacious NF-κB inhibitors to block NF-κB activity, which could also benefit BCa patients with IL-1-NF-κB-driven disease.

## Figures and Tables

**Table 1:** Anakinra cancer clinical trials.

Clinical Trial-#	Phase	Cancer Type	Disease Characteristics	Treatment	Start Date	Completion Date/Status	Published Results	Reference
NCT00635154	II	Multiple Myeloma and Plasma Cell Neoplasm	Indolent or Smoldering Multiple Myeloma (asymptomatic)	Anakinra in combination with dexamethasone	2002	2010	Improved progression free survival	[99]
NCT00072111	I	Unspecified solid tumors	Progressive metastatic cancer non-responsive to chemotherapy with tumor expression of IL-1	Anakinra tolerability	2003	2006		
NCT01802970	I	Breast Cancer	Locally unresectable, invasive, or metastatic	Anakinra in combination with nab-paclitaxel, capecitabine, eribulin, or vinorelbine	2012	2017	Pilot study results, n = 11 patients; 2 = tumor volume reduction, 4 = stable disease, 2 = stopped anakinra for injection site reaction, 3 = progressive disease. Reduction in systemic IL-1 transcriptional signature.	[11]
NCT01624766	I	Advanced/metastatic cancers	Non-responsive to standard therapy	Everolimus in combination with anakinra or denosumab	2012	2021		
NCT02021422	I	Pancreatic cancer	Inoperable, metastatic	Anakinra in combination with oxaliplatin, Irinotecan, or fluorouracil	2013	2017		
NCT02090101	II	Colorectal Cancer	Metastatic, non-responsive to chemotherapy	Anakinra in combination with LV5FU2 and bevacizumab	2014	2017	Combination therapy was tolerated and increased overall survival	[100]
NCT02550327	I	Pancreatic Cancer	Suspected prior to diagnosis or histologically diagnosed pancreatic cancer	Anakinra in combination with three-drug regimen of nab-paclitaxel, gemcitabine, and cisplatin	2016	2021		
NCT02492750	I	Plasma Cell Myeloma	Indolent or Smoldering Plasma Cell Myeloma (asymptomatic)	Anakinra in combination with lenalidomide and dexamethasone	2016	2019		
NCT03233776	II	Multiple Myeloma	Diagnosed with multiple myeloma, scheduled to receive an autologous stem cell transplantation fter myeloablative therapy with high-dose melphalan	Anakinra in combination with autologous stem cell transplantation and melphalan	2017	2020		
NCT03430011	II	Multiple Myeloma	Relapsed and/or refractory disease, non-responsive to autologous stem cell transplant, immunomodulatory agents, proteosome inhibitors, and anti-CD38	Anakinra in combination with JCARH125 (CAR-T that targets B-cell maturation antigen)	2018	estimated, 2023		
NCT04099901	II	Multiple Myeloma	Diagnosed with multiple myeloma, scheduled to receive an autologous stem cell transplantation fter myeloablative therapy with high-dose melphalan	Anakinra in combination with autologous stem cell transplantation and melphalan [placebo added]	2019	estimated, 2022		
NCT04227275	I	Metastatic Castration Resistance Prostate Cancer	At least 2 prior lines of systemic therapy, including second generation androgen receptor inhibitor and/or CYP17α inhibitor	Anakinra in combination with cyclophosphamide and fludarabine lymphodepletion and CART-PSMA-TGFβRDN	2019	estimated, 2036	Initial observations indicate immune toxicity management strategy needed; prophylactic anakinra instituted.	[101]
NCT04150913	II	B-cell Lymphoma	Relapsed or refractory large B-cell lymphoma after two or more lines of systemic therapy	Anakinra In combination with axicabtagene ciloleucel	2020	estimated, 2024; recruiting		
NCT04432506	II	B-cell Lymphoma	Relapsed or refractory B-cell lymphoma, at least 2 prior lines of systemic therapy	Anakinra in combination with axicabtagene ciloleucel, cyclophosphamide, and fludarabine	2020	estimated, 2025; recruiting		
NCT04691765	I	Chronic Lymphocytic Leukemia	Diagnosis of Chronic Lymphocytic Leukemia (CLL) meeting published diagnostic criteria, not currently treated with other agents for CLL.	Anakinra	2021	estimated, 2022; not yet recruiting		
NCT04942626	I	Rectal Cancer	Localized	Anakinra in combination with capecitabine and radiation	2021	estimated, 2026; not yet recruiting		
NCT04926467	II	Pancreatic Cancer	Resectable, locally advanced or potentially resectable pancreatic adenocarcinoma	Anakinra in combination with pre-operative nab-paclitaxel, gemcitabine and cisplatin and post-operative 5-fluorouracil, oxaliplatin, and irinotecan	2021	estimated, 2026; not yet recruiting		

## Data Availability

Not applicable.
